# A Case Illustrating the Importance of Diagnosis

**Published:** 1882-08

**Authors:** H. R. Hopkins


### A Case Illustrating the Importance of Diagnosis.

By H. R. Hopkins, M. D.

The following case illustrating the importance of diagnosis, may be of interest. It may be said that it is not all necessary to enforce the application of a principle, which commands the ready assent of every member of the profession, and upon the subject I would like to say, that it seems to me, that we are quick enough to admit the general principle, but that many of us are very remiss in putting this principle into action in our individual cases. Among our busy men, how many will be found who hold themselves accountable for making an exhaustive examination, and an exact diagnosis in each case that comes under their observation, is not the habit quite too common of making just sufficient examination to enable one to exclude certain formidable troubles, which might stand as a cause of the symptoms presented, coupled with the further habit of ordering an expectant treatment, and then waiting in a lazy way for time to clear the obscurities. My opportunities for observation upon the habits of some of our leading men, by no means the chief of sinners, forces me to consider this one of the mental habits we should constantly strive to avoid. Aside from the wrong we do our patients, by not giving them the advantage of an intelligent and exact diagnosis, and a suitable treatment and prognosis, which can only be predicated upon such a diagnosis, we commit even a greater wrong upon ourselves by allowing the mind to get into that lazy, misty emasculate condition, in which vigorous, virile, constructive thought becomes impossible.

Is it not true that the real hold the medical profession, as opposed to the empirics, have upon the confidence of the public, is in the power of early and intelligent diagnosis and prognosis ?

These thoughts, perhaps very crude and inconsequential, were called to my mind by a fortunate diagnosis, the result of accident which I made upon the following case.

Was called to see J. B., aged 73, laborer, in the forenoon of the 1st inst. He had worked as usual the previous day, which had been quite warm and he had perspired quite freely and drank an unusual quantity of ice water. Went to bed as usual and was wakened in the night with pain in the abdomen, about the navel, soon followed by copious diarrhœa and vomiting. Had six or eight liquid stools with considerable tenesmus.

About daylight the diarrhœa ceased, but the vomiting still continued, attacks coming about every twenty minutes. He was in bed with a cool, moist skin; normal heat and pulse; no pain; a little tender over the belly. I made in advance a diagnosis a mild gastro-enteritis, but proceeded to a more thorough examination, as the man had called at my office a fortnight before, complaining of swelling of his feet, and I thought examination would reveal a diseased liver.

Upon laying bare the belly, I saw a lump as big as half a hen's egg in the right groin, about the middle, and just below Poupart's ligament. This moved easily under the skin, which was free from discoloration, was not tender nor painful. He thought the lump was not there the day before, that it must have come in the night, and was sure it was of no account.

Upon close examination I found that, while the lump seemed to move under the fingers quite freely, this movement was confined to its more prominent or superficial portion; that it had a base quite deeply seated and firmly fixed. Upon once more interrogating the patient and his attendants, I found that the vomit for the few last times had a very strange odor, almost like the stool. The diagnosis of strangulated femoral hernia was made and ice applied to the lump. As soon as it could be arranged, about three hours, Dr. Folwell saw the case in consultation and confirmed the diagnosis. The arrangements for operating were made, when the formal employment of taxis was observed, much to our surprise with entire success. The relief of all symptoms was immediate and complete, and on the 4th of July, three days afterwards, I saw him in our procession with a banner, saying that "he had worked for the same person for over 32 years."

Here was a case in which the data for diagnosis were present and ample. In fact, the danger of not making a correct diagnosis came from the profusion of symptoms. Without the observation of all the facts the diagnosis of gastro-intestinal irritation would, undoubtedly, have held long enough to have lost the chance of saving the man's life.

With this erroneous diagnosis, once put into effect, the chance of correcting the error would rest mainly upon the opportunity to observe the nature of the vomit, which might not occur in at least 24 or 48 hours. If the odor was simply suspicious, the suspicion would be dismissed in view of the history of diarrhœa, making a strong presumption against a hernia Without the knowledge of the presence of the lump in the groin, the symptoms arranged themselves in perfect order about the idea of gastro-intestinal irritation, while, with that knowledge, they grouped just as handily about a quite dissimilar cause and thus formed an altogether different case. If one had not made the diagnosis by accident, they would be tempted to point out the helplessness of the empirical method of treating symptoms in the face of this and similar cases.

